# The Emerging Role of Senolytics as a Next-Generation Strategy Against Glioma Recurrence: A Narrative Review

**DOI:** 10.3390/cancers18081220

**Published:** 2026-04-12

**Authors:** Andrea Filardo, Isabella Coscarella, Jessica Bria, Anna Di Vito, Domenico La Torre, Emanuela Chiarella, Adele Giovinazzo, Emanuela Procopio, Maria Teresa Egiziano, Angelo Lavano, Attilio Della Torre

**Affiliations:** 1 Department of Medical and Surgical Sciences, University Magna Graecia of Catanzaro, 88100 Catanzaro, Italy; filardo@unicz.it (A.F.); isabella.coscarella@studenti.unicz.it (I.C.); jessica.bria@studenti.unicz.it (J.B.); emanuelachiarella@unicz.it (E.C.); adele.giovinazzo@studenti.unicz.it (A.G.); emanuela.procopio@studenti.unicz.it (E.P.); mariateresa.egiziano@studenti.unicz.it (M.T.E.); a.dellatorre@unicz.it (A.D.T.); 2 Department of Clinical and Experimental Medicine, University Magna Graecia of Catanzaro, 88100 Catanzaro, Italy; divito@unicz.it; 3 Neurological Unit, Department of Pharmacy, Health and Nutrition Sciences, University of Calabria, 87036 Rende, Italy; domenico.latorre@unical.it

**Keywords:** senescence, glioma, cancer therapy, glioblastoma, senolytics

## Abstract

Cellular senescence represents a critical biological paradox in oncology. Although it evolved as a safety mechanism to halt tumorigenesis through stable cell cycle arrest, its persistence in tissues can alter the microenvironment, promoting tumor recurrence. In the context of glioblastoma (GBM), this phenomenon is critically important, as current standard therapies, such as radiotherapy and chemotherapy, inadvertently induce a state of senescence known as “therapy-induced senescence” (TIS). Senescent cells remain metabolically active and acquire a unique Senescence-Associated Secretory Phenotype (SASP), characterized by the release of pro-inflammatory cytokines, proteases, and growth factors. SASP reshapes the tumor microenvironment (TME) through paracrine signals, promoting immunosuppression, invasiveness, drug resistance and tumor recurrence. Different glial populations, including astrocytes, microglia, and oligodendrocyte precursor cells (OPCs), respond differently to senescence, specifically contributing to the creation of a permissive niche for tumor recurrence. To contrast the effects of this phenomenon, a promising therapeutic strategy has emerged, the “one-two punch,” which induces initial DNA damage followed by selective elimination of senescent cells with senolytic drugs. In this review, we analyze in detail the efficacy of targeted synthetic agents, such as the *Bcl-2* family inhibitor Navitoclax, and natural bioactive compounds such as Quercetin and Fisetin. The analysis focuses on the molecular mechanisms through which these agents disrupt anti-apoptotic pathways (SCAPs) and inhibit the PI3K/AKT/mTOR axis, restoring sensitivity to apoptosis. We propose that the integration of senolytic adjuvants into standard clinical protocols may represent a crucial frontier for eliminating residual disease reservoirs and we also suggest the possibility of combining them with molecules with neuroprotective action to significantly improve the prognosis in GBM.

## Simple Summary

Standard glioblastoma (GBM) treatments inadvertently induce therapy-induced senescence (TIS). While halting immediate tumor growth, these senescent cells secrete a pro-inflammatory Senescence-Associated Secretory Phenotype (SASP) that remodels the microenvironment, ultimately driving immunosuppression, resistance, and disease recurrence. To resolve this clinical paradox, this review evaluates the emerging “one-two punch” strategy: leveraging conventional therapies to drive tumor cells into senescence, followed by targeted senolytic drugs to dismantle their survival mechanisms and force apoptosis. Because clearing senescent cells can trigger local inflammation, we propose a novel integrated approach to mitigate the potential neurotoxicity and inflammation caused by the therapeutic clearance of these cells, combining senolytic regimens with natural neuroprotective agents.

## 1. Introduction

The management of GBM remains one of the most daunting challenges in modern neurooncology. Despite aggressive multimodal treatments, which typically include maximal surgical resection followed by concurrent radiation therapy and chemotherapy with temozolomide (TMZ), the prognosis remains poor. Although ionizing radiation and alkylating agents effectively inflict lethal DNA damage on rapidly dividing cells, they also force a significant fraction of surviving tumor and stromal cells into a state of stable cell cycle arrest, known as TIS. Therefore, this manuscript aims to provide a perspective by evaluating not only the efficacy of senolytic agents in dismantling the tumor’s SASP, but also the unprecedented necessity of coupling these agents with neuroprotective strategies. By critically analyzing the current translational bottlenecks—such as optimal therapeutic timing, hit-and-run dosing, and patient stratification—this review seeks to outline a more holistic and clinically sustainable “one-two punch” paradigm for neuro-oncology.

### 1.1. Cellular Senescence

Cellular senescence represents a state of stable and essentially irreversible cell cycle arrest. Distinct from quiescence or terminal differentiation, this process acts as a critical fail-safe against tumorigenesis [1,2,3]. By locking damaged cells into this state, the body prevents those suffering from oncogenic stress or significant injury from undergoing malignant transformation [4,5,6]. Various stressors can trigger this response. These range from internal factors like mitochondrial deterioration, genotoxic stress (DNA damage and chromatin alteration), and telomere dysfunction, to external insults including chemo- and radiotherapy. Even a spike in reactive oxygen species or the activation of specific oncogenes can force a cell into senescence [7,8,9,10,11]. Ultimately, this mechanism serves as a vital, cell-autonomous brake, suppressing tumor growth in its earliest developmental stages [4,5,12].

### 1.2. The Paradox of Cellular Senescence

While cellular senescence serves as a vital tumor suppressor in early stages, recent evidence highlights a paradox: its persistence can eventually aid the enemy. We now understand that when senescent cells accumulate, either through natural aging or cytotoxic treatments (Therapy-Induced Senescence, TIS), they create a microenvironment that fuels tumor progression, metastasis, and drug resistance [1,12,13,14]. This duality makes the pathway an attractive therapeutic target. Crucially, although these cells lose the ability to divide, they remain metabolically active, adopting a complex secretory profile; furthermore, they exert powerful paracrine effects on neighboring cells, effectively remodeling the Tumor Microenvironment (TME) to favor malignancy [4,14].

### 1.3. Molecular Mechanism

Entry into senescence is governed by a multilevel regulatory network, primarily driven by the p53/p21^CIP1^ and p16^INK4A^/Rb axes. These two pathways can operate independently, coordinate their efforts, or function as redundant fail-safes depending on the context [5,11].

#### 1.3.1. The p53/p21^CIP1^ Pathway

Typically triggered by acute genotoxic stress such as ionizing radiation, oxidative damage, or telomere dysfunction, this pathway begins with the activation of ATM (Ataxia-telangiectasia mutated) and ATR (ATM and Rad3-related) kinases, two master transducers of DNA damage signals. These enzymes stabilize the transcription factor p53, which in turn drives the expression of p21, a potent inhibitor of cyclin-dependent kinases (CDKs). By blocking CDK activity, p21 prevents Rb phosphorylation; this keeps E2F transcription factors suppressed and halts the cell cycle at the G1/S checkpoint. While this pause initially offers a window for DNA repair, persistent or irreparable damage converts the temporary arrest into permanent senescence [15,16].

#### 1.3.2. The p16^INK4A^/Rb Pathway

In contrast, the p16INK4A pathway is often more distinct in its triggers, responding primarily to chronic stress, oncogenic signalling, or cumulative telomere erosion. Here, p16 directly inhibits CDK4 and CDK6 kinases. This blockade maintains Rb in a hypophosphorylated, active state, enabling it to sequester E2F factors and effectively silence the genes required for DNA synthesis. Once fully engaged, this pathway almost invariably locks the cell into an irreversible senescent state [17,18].

### 1.4. Senescence Associated Secretory Phenotype (SASP)

**Figure 1:**
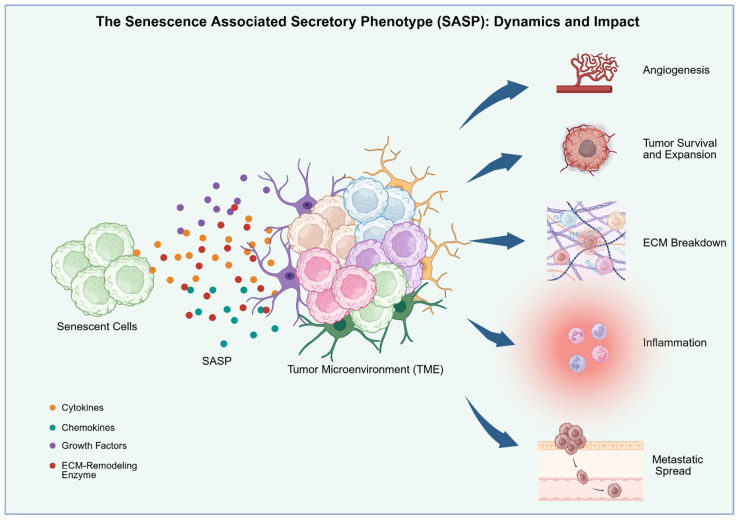
SASP dynamics and impact. Senescent cells secrete a wide range of bioactive molecules—including pro-inflammatory cytokines, chemokines, growth factors, and matrix metalloproteinases—collectively known as the Senescence-Associated Secretory Phenotype (SASP). These factors exert their effects through autocrine and paracrine signaling pathways, modulating inflammation, angiogenesis, and extracellular matrix dynamics within the tissue microenvironment.

Beyond the defining feature of stable cell cycle arrest, senescent cells exhibit distinct morphological and metabolic shifts, alongside a significant resistance to apoptosis. However, the hallmark of this state is undoubtedly the acquisition of the Senescence-Associated Secretory Phenotype (SASP). This phenotype functions as a biological paradox; indeed, while it facilitates immune recruitment and tissue repair, the secretion of specific pro-inflammatory factors can trigger detrimental outcomes. Structurally, the SASP represents a dynamic and complex secretome; indeed, it comprises a vast array of cytokines (such as IL-6 and IL-8), chemokines, growth factors like VEGF, and enzymes involved in extracellular matrix (ECM) remodeling, specifically matrix metalloproteinases (MMPs). SASP composition alters significantly under different microenvironmental conditions both spatially and temporally. Changes in SASP composition occur along a continuum, dependent on the cellular and non-cellular components of the tissue microenvironment. Similarly, a different SASP composition is evident in early, intermediate and late senescence. Operating through both autocrine and paracrine signalling, SASP may drive proliferation, angiogenesis, and inflammation [19,20,21,22]. Unlike the acute effects of SASP, chronic activation may serve as the main driver of pro-tumorigenic activity by releasing a potent cocktail of bioactive molecules that support tumor cell survival and expansion. For example, MMPs break down the extracellular matrix to enable metastatic spread, whereas growth factors like VEGF fuel angiogenesis. Through these mechanisms, showed in Figure 1, the senescent secretome can effectively corrupt a tissue niche, converting it into an environment that is pro-inflammatory, pro-angiogenic, and pro-invasive [1,13,14]. Key regulators controlling the SASP include NF-κB, p53, C/EBP, and GATA4. Specifically, NF-κB and C/EBPβ function as the transcriptional engines for pro-inflammatory cytokines and chemokines. Furthermore, GATA4 amplifies NF-κB activity via the secretion and expression of IL-1A. In contrast, p53 modulates the secretory profile qualitatively; it attenuates or remodels the SASP, shifting it toward p53-dependent factors (P-SASP) and curbing pro-inflammatory cytokine expression by inhibiting the p38MAPK–NF-κB axis [9,21,22,23].

### 1.5. The Dynamic and Context-Dependent Nature of Senescence

Cellular senescence is a process characterized by high plasticity and strong context dependency, in which the final phenotypic outcome is modulated by a complex interplay of temporal, spatial, and cell type-specific variables. The temporal axis governs the transition from physiological to pathological senescence. Acute senescence is typically a transient and beneficial response; it halts the proliferation of damaged cells and employs its early senescence-associated secretory phenotype (SASP) to actively recruit immune effectors such as macrophages and Natural Killer (NK) cells, thereby facilitating tissue repair and the subsequent immune clearance of senescent cells.

Conversely, when immune surveillance is overwhelmed, or when senescence is induced massively and simultaneously within a tissue—such as following genotoxic therapies (therapy-induced senescence, TIS)—the state becomes chronic. This prolonged persistence allows the secretome to evolve, progressively remodeling the tissue microenvironment to promote local immunosuppression and drive malignant phenotypes [24,25,26].

Moreover, the senescence program is profoundly influenced by the cell of origin. The specific nature of the triggering stressor and the cellular lineage (e.g., epithelial, stromal, or immune) dictate distinct SASP compositions and metabolic adaptations [27]. In this context, mapping the dynamic molecular profile of the microenvironment becomes crucial; for instance, analyzing combined patterns of metabolic stressors like glycation adducts and cytokines in the secretome of short-term blood-derived cultures has proven valuable in reflecting these complex shifts during tumor progression [28]. Consequently, the net biological effect of senescence is not an intrinsic, static property, but rather a dynamic state determined by the duration of cell cycle arrest and the unique microenvironmental niche, thereby laying the groundwork for the complex heterotypic interactions observed in challenging tumors such as gliomas.

### 1.6. Epigenetic Mechanisms Regulating Cellular Senescence

The stability of the senescent state in glioblastoma and the production of the SASP are not simply driven by kinase signaling cascades but are rooted in profound epigenetic and chromatin remodeling. Entry into senescence is typically accompanied by the formation of senescence-associated heterochromatin foci (SAHF) and the loss of the nuclear protein LMNB1, which alters the three-dimensional architecture of the genome [29,30].

At the histone level, senescent glioma cells exhibit a reprogrammed landscape characterized by a global loss of the repressive mark H3K27me3 and a focal accumulation of H3K9me3. This specific epigenetic signature exerts a dual effect: on the one hand, it irreversibly silences gene loci essential for cell cycle progression; on the other, it maintains an open and accessible chromatin architecture at the promoters of pro-inflammatory genes such as IL-6 and TGF-β, thereby actively sustaining the SASP [29,30].

## 2. Senescence in the Clinical Context of Gliomas

In the landscape of oncology, gliomas represent a heterogeneous class of highly aggressive primary brain tumors in adults [31]. The current standard of care employs a multimodal approach involving chemotherapy, radiotherapy and surgical resection, the latter now frequently enhanced by intraoperative neurophysiological monitoring [32]. Recently, Chimeric Antigen Receptor T-Cell Therapy (CAR-T) has attracted considerable interest as a potential breakthrough, although it is still in early stages of clinical validation for gliomas [33]. Among these therapeutic options, radiotherapy (RT) remains a key choice; it typically provides only transient local control, extending progression-free survival to a window of approximately 6–9 months. Consequently, the overall prognosis for glioma patients, particularly those diagnosed with glioblastoma (GBM), remains poor, characterized by extremely high mortality rates and a life expectancy of approximately 15 months [34].

### Limitations of Current Therapies and Therapy-Induced Senescence (TIS)

Accumulating data identifies cellular senescence as a pivotal mechanistic driver of therapeutic failure and disease recurrence. Historically, the literature has prioritized the intrinsic adaptive mechanisms of tumor cells such as drug resistance, autophagy, Glioblastoma Stem Cell (GSC) maintenance, and hypoxic responses; however, this focus has often overlooked the profound remodeling effects that RT exerts on the TME. The central goal of current standard-of-care therapies is the elimination of tumor bulk via cytotoxic means, primarily using ionizing radiation or DNA alkylating agents like temozolomide (TMZ). Yet, this approach comes with a cost: while effective, the resulting DNA damage triggers stable cell cycle arrest, forcing cells into a state of Therapy-Induced Senescence (TIS) [35,36]. Despite being proliferatively inert, these cells remain metabolically active and highly resistant to apoptosis; additionally, they develop a robust SASP capable of negatively impacting the surrounding tissue. Specifically, TMZ-driven senescence activates the SASP via an NF-κB-dependent pathway; it represses the transcription of essential mismatch repair and homologous recombination proteins, including EXO1, MSH2, MSH6, and RAD51-59 [37]. The existence of these senescent populations marked by specific proteins like p16INK4A has been validated in both human biopsy samples and murine GBM models. Notably, Salam et al. demonstrated that the partial clearance of malignant senescent cells in a mouse model of GBM, significantly altered the tumor ecosystem and improved the survival of tumor-bearing mice. While their model was genetically distinct from human patient profiles, it successfully replicated key histopathological features, including cell states heterogeneity and the senescent traits observed in clinical cases [11]. These senescent cells effectively act as a reservoir for residual disease; supported by scientific evidence, they retain the capacity to escape the senescent state, re-enter the cell cycle, and ignite tumor recurrence. The frequent recurrence of glioma in peritumoral regions subjected to high radiation doses suggests that irradiated stromal cells may cultivate a niche favorable for GBM survival and re-expansion [11]. Furthermore, emerging data indicates that radiation-induced senescent stromal cells exert pro-tumorigenic effects via paracrine signaling. For instance, pre-irradiated mice exhibit reduced survival following intracranial GBM injection, reinforcing the hypothesis that TIS actively primes the TME for recurrence [5].

## 3. Cellular Heterogeneity and SASP-Mediated Mechanisms of Progression

Post-irradiation senescence is not a uniform phenomenon. Recently, the heterogeneity of GBM cells has been characterized by single-cell RNA sequencing approaches, highlighting four main phenotypes: oligodendrocyte progenitor cell-like (OPC-like), neural progenitor cell-like (NPC-like), astrocyte-like (AC-like), and mesenchymal-like (MES-like) states [38]. Besides tumor cells, other cell populations also contribute to tumorigenesis, including immune cells, endothelial cells, microglia, as well as astrocytes and neurons. Both tumor and non-tumor cells can equally develop irradiation-induced senescence; however, the resulting Senescence-Associated Secretory Phenotype (SASP) differs significantly across cell types [39], generating a complex web of signals within the TME [40].

### 3.1. Senescent Astrocytes (SnAs)

Recently, the accumulation of radiation-induced senescent astrocytes (SnAs) has been recognized as a key driver of GBM progression. Molecularly, these cells evolve into hyper-secretors of CXCL12, G-CSF, TNF-α, sICAM-1, and IL-6. Crucially, astrocytic TNF-α drives CXCL1 expression in GBM cells by activating the c-Myc–Max transcriptional complex. This signalling axis favors the recruitment of CXCR2+ cells, effectively fostering an immunosuppressive TME [41]. Furthermore, using murine models, Fletcher-Sananikone et al. mapped a specific paracrine loop: following irradiation, SnAs secrete elevated levels of Hepatocyte Growth Factor (HGF). As a key SASP component, HGF binds and activates its receptor tyrosine kinase (RTK), Met, on the glioma cell surface. This HGF/Met interaction triggers a signaling cascade that serves as a potent stimulus for tumor growth, migration, and invasion (Table 1) [5].

### 3.2. Oligodendrocyte Precursor Cells (OPCs)

Emerging evidence suggests that senescent OPCs display SASP profiles distinct from those of astrocytes, reflecting their physiological role in myelination. These cells appear to influence tumor progression through two main avenues: modulating chemoresistance via cytokines like EGF and FGF1, and reshaping the vasculature and microglial landscape through the downregulation of TGFβ signaling. These findings indicate that OPCs function as complex biological switches, generating opposing signals depending on their state (senescent vs. non-senescent)—a dichotomy whose full biological outcomes remain to be defined (Table 1) [41].

### 3.3. Microglia

Radiation exposure forces microglia into a distinct senescence-like, pro-inflammatory phenotype. This transition is orchestrated by the activation of NF-κB and mitogen-activated protein kinase (MAPK) pathways, resulting in a surge of cytokine secretion. This secretory profile exerts a dual, context-dependent effect: on the one hand, the inflammatory surge causes neurotoxicity and neuronal apoptosis [42]. On the other hand, within the tumor niche, this chronic SASP—characterized by upregulated signatures of IL-6, IL-8, CCL2, TNF-α, and TGF-β—paradoxically fosters an immunosuppressive TME, protecting the tumor from immune surveillance (Table 1) [41].

### 3.4. The IL-6/JAK/STAT3 Pathway

**Figure 2:**
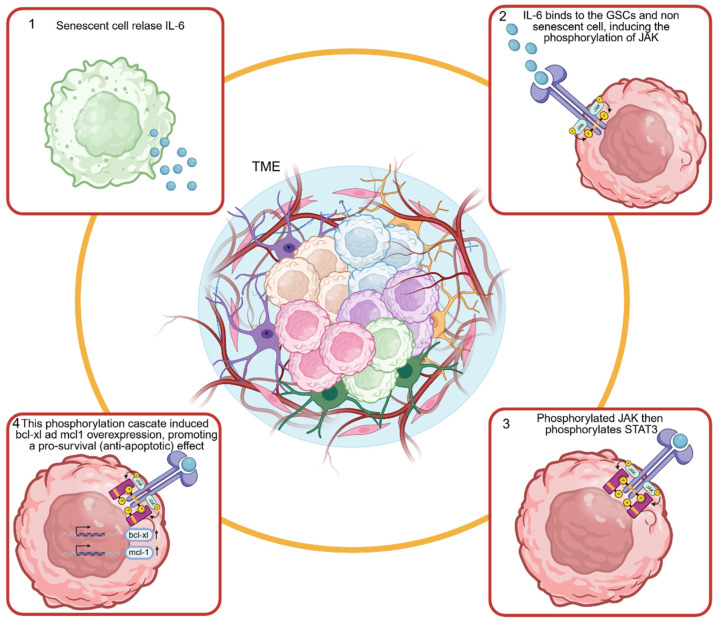
The role of the IL-6/JAK/STAT3 axis in tumor progression. The acquisition of the senescence-associated secretory phenotype (SASP) triggers the release of pro-inflammatory cytokines, notably IL-6, which activates the JAK/STAT3 signaling cascade in neighboring tumor cells and glioblastoma stem cells (GSCs). JAK phosphorylation leads to the constitutive activation of STAT3, which in turn upregulates the expression of anti-apoptotic genes (*Bcl-xL* and *Mcl-1*) and drives metabolic reprogramming toward aerobic glycolysis. Collectively, these processes enhance cell survival, tumor aggressiveness, radioresistance, and recurrence. Arrows indicate an increase of *bcl-xl* and *mcl-1*.

The acquisition of the SASP—whether in tumor or non-tumor cells—induces the release of mediators able to activate key pro-survival pathways. Among these, the IL-6/JAK/STAT3 axis is widely recognized as the master pathway in senescence-induced tumorigenesis [43,44]. Specifically, this cascade serves as the critical functional bridge linking cellular senescence to the maintenance of Glioblastoma Stem Cells (GSCs) [45] and the acquisition of radioresistance [46]. Central to GBM progression is the crosstalk mediated by senescent cell-derived IL-6 and the subsequent engagement of the JAK/STAT signaling cascade. Upon secretion, IL-6 binds to receptor complexes on adjacent non-senescent tumor cells and Glioblastoma Stem Cells (GSCs); this interaction triggers JAK phosphorylation, which in turn drives the constitutive activation of STAT3 [47]. The IL-6/JAK/STAT3 axis acts as a survival engine; indeed, it upregulates key anti-apoptotic genes, specifically *Bcl-xl* and *Mcl-1*, and orchestrates a metabolic shift toward aerobic glycolysis (Warburg Effect) as showed in Figure 2. These adaptations confer a phenotype of heightened tumor aggressiveness, therapeutic resistance, and inevitable recurrence [44,48].

### 3.5. Biological Variables: The Sex-Specific Role of p21

While p21 is established as a central mediator of irradiation-induced senescence, its correlation with the classical marker SA-β-gal diverges significantly according to biological sex. Data indicates that at equivalent p21 thresholds, female cells manifest a more profound senescent phenotype compared to their male counterparts, pointing to a heightened female sensitivity to p21 signaling. Further dissection using the Four Core Genotypes (FCG) model of GBM reveals that this disparity is primarily driven by gonadal sex. This strongly suggests that the hormonal milieu plays a critical role in modulating the p21-mediated senescent response [49]. Clinically, this mechanism helps explain the established sexual dimorphism in GBM prognosis. In females, robust p21 activation effectively halts the cell cycle of damaged cells (senescence as tumor suppression); in contrast, male cells—prone to bypassing this checkpoint due to lower p21 sensitivity—sustain rapid tumor expansion and earlier recurrence [50,51].

### 3.6. Mechanisms of Senescence Escape and Implications for Tumor Recurrence

Despite senescence being traditionally considered a stable cell cycle arrest, recent evidence indicates that, in the context of glioblastoma, therapy-induced senescence (TIS) often functions as a state of transient dormancy, in which residual tumor cells remain viable and metabolically active after treatment, thereby constituting a potential reservoir for recurrence. Through a process of genomic reprogramming, a subpopulation of senescent cells can escape cell cycle arrest (senescence escape) [52]. From a mechanistic perspective, this escape is mediated by endoreplication events (leading to the formation of polyploid giant multinucleated cells, PGCCs) and the subsequent downregulation of cell cycle checkpoints, such as p21 and p53. The clinical consequence of this “reawakening” is particularly detrimental as cells that re-enter the cell cycle exhibit a profoundly altered transcriptional profile, characterized by increased stemness, phenotypic plasticity, and acquired chemoresistance [53]. The use of senolytic agents therefore finds its strongest biological rationale not only in the elimination of the SASP, but also in the need to eradicate this dormant reservoir of tumor cells before they can trigger disease recurrence.

## 4. Senolytic Drugs: Strategies and Mechanisms

The cornerstone of senolytic therapy is the targeted ablation of senescent cells; however, given the marked heterogeneity of brain tumors, the selection of highly specific agents is crucial. Preclinical evidence suggests that the use of these drugs as adjuvants to standard GBM protocols could be promising strategy to significantly reduce relapses. This therapeutic paradigm, often referred to as the “one-two punch,” operates through a sequential biphasic approach [54,55]. The initial phase employs standard cytotoxic modalities, such as chemotherapy or radiation therapy, to reduce tumor mass. Ultimately, this halts the proliferation of surviving cells, forcing them into a state of TIS [55,56]. The second phase is designed to selectively eliminate this TIS population, thereby eliminating the primary source of SASP and neutralizing the pro-tumorigenic microenvironment that fuels recurrence [36]. Evidence from mouse models confirms that pharmacogenetic or pharmacological clearance of senescent cells significantly prolongs survival and favorably reshapes the tumor immune microenvironment (TIME) [5,11]. However, some caveats are needed: senolytic agents such as ABT263 and Dasatinib plus Quercetin (D+Q) combination trigger different modes of cell death, and the sensitivity of GBM cells to these drugs varies depending on both the cell line and the post-irradiation state [57].

### 4.1. SCAPs and the Classification of Senolytics

Given this molecular heterogeneity and variable response, classifying senolytics based on senescent cell survival mechanisms is crucial. These cells resist apoptosis by activating specific pro-survival networks, collectively known as senescent cell anti-apoptotic pathways (SCAPs) [57]. The main identified SCAPs include the *BCL-2* family-mediated mitochondrial axis, the PI3K/AKT/mTOR pathway, the p53/p21 regulatory network, and pro-survival signals driven by tyrosine kinase receptors [58]. Senolytics work by selectively shutting down one or more of these survival networks, restoring the senescent cells’ vulnerability to apoptosis. Because of this, we can classify these agents not just as individual compounds, but into mechanistic classes based on the pathway they target [57,59]. Pharmacologically, they fall into synthetic agents—like *BCL-2* family and tyrosine kinase inhibitors—and natural compounds. Natural compounds, such as quercetin and fisetin, typically exert a pleiotropic action across multiple SCAPs [59]. In the context of GBM, however, these anti-apoptotic pathways are not equally relevant across different tumor subpopulations. Preclinical studies mostly zero in on molecular circuits known to be overexpressed or functionally active in glioma, such as the *BCL-2* axis and PI3K/AKT signaling [60,61]. Similarly, researchers have only looked into a limited number of senolytics in this setting so far. In some cases, practical factors drove their selection rather than just mechanistic reasoning. These include clinical availability, a known safety profile, and drug-repurposing potential, as seen with the D+Q combination [57].

The following sections map out the senolytics studied in GBM. We organize them by their target SCAPs and sort them into synthetic agents and natural compounds (Table 2).

### 4.2. Bcl-2 Family Inhibitors

**Figure 3:**
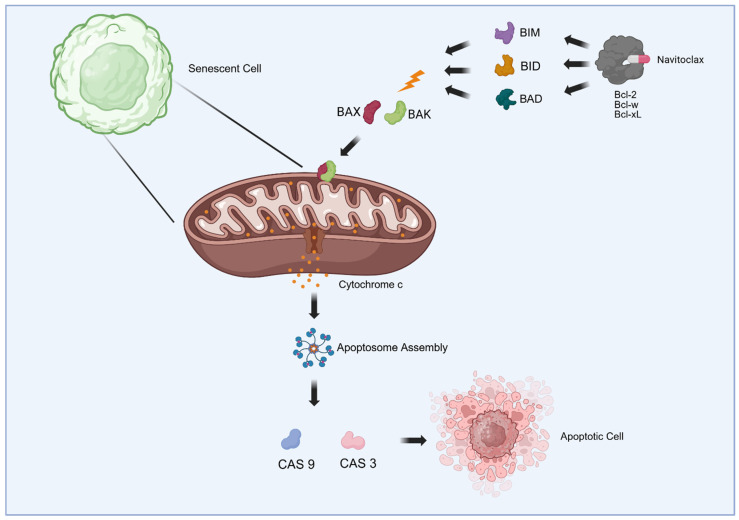
Mechanism of action of navitoclax in senescent cells. Senescent cells exhibit a robust dependency on anti-apoptotic proteins of the *BCL-2* family. Navitoclax, a BH3-mimetic, binds to *Bcl-2*, *Bcl-xL*, and *Bcl-w*, thereby displacing pro-apoptotic proteins and triggering the intrinsic apoptotic cascade. This process leads to mitochondrial outer membrane permeabilization, the release of cytochrome c and caspase activation, resulting in the selective clearance of senescent cells.

Given that senescent cells exhibit a critical dependence on anti-apoptotic pathways for survival, the primary strategy for the development of senolytic drugs is to disrupt these survival signals. Proteomic analyses have confirmed the overexpression of key family members, particularly *BCL-2*, *BCL-xL*, *BCL-w*, and *MCL-1*, along with activation of the PI3K-AKT axis in senescent populations. In particular, GBM are characterized by the overexpression of these anti-apoptotic proteins [62,63]. Consequently, preclinical efforts have primarily focused on ABT-263, a potent inhibitor of *Bcl-2*, *Bcl-xL*, and *Bcl-w*. Mechanistically, navitoclax mimics the BH3 domain, binding with high affinity to the hydrophobic groove of *Bcl-2*/*Bcl-xL*. This binding displaces pro-apoptotic effectors such as Bim, Bid, and Bad, thereby triggering the intrinsic apoptotic cascade, as illustrated in Figure 3. The resulting sequence includes mitochondrial outer membrane permeabilization (MOMP), cytosolic release of cytochrome c, apoptosome assembly, and the execution of cell death via caspases 9 and 3 (Table 2) [64,65]. In vitro studies using a broad range of grade IV astrocytoma cell lines, including LN229, A172, and U87MG, as well as primary and recurrent cultures of human P53 and IDH-WT cells, demonstrate that navitoclax induces apoptosis in senescent GBM cells with significantly greater potency (reflected in lower IC50 values) compared to non-senescent controls. However, sensitivity to *Bcl-xL* inhibition is inconsistent; it varies among cell lines and is influenced by radiation status [5]. In vivo, treatment with ABT-263 in pre-irradiated models successfully promoted the ablation of senescent cells, correlating with reduced aggressiveness of GBM implants in the irradiated brain. In addition to direct cytotoxicity, targeting *Bcl-2* proteins could provide a secondary benefit: by eliminating the cellular source of inflammatory signaling, these inhibitors could attenuate the pro-tumorigenic microenvironment and mitigate recurrence [5]. Despite its efficacy, the clinical translation of Navitoclax remains hampered by dose-limiting toxicities (primarily thrombocytopenia) required for prolonged treatment. This has spurred interest in more selective agents with similar apoptotic potential, such as the specific *Bcl-xL* inhibitors A1331852 and A1155463 [66].

### 4.3. The Effects of Navitoclax on p21-Dependent Senescence in GBM

Recent preclinical studies point out a specific vulnerability in the GBM landscape: cellular senescence can leave tumor cells highly susceptible to p21-dependent senolytic treatments in vitro. Specifically, Niklasson et al. showed that turning on the bone morphogenetic protein 4 (BMP4) signaling pathway—a differentiation-inducing factor explored as a therapeutic strategy to cut down stemness and tumor growth—triggers a senescence program mostly driven by p21 [67]. BMP4 treatment brings about phenotypic alterations and turns on senescence-associated genes, such as cellular hypertrophy, increased SA-β-gal activity, a drop in lamin B1, and a sharp upregulation of p21 [67]. Importantly, this response sensitivity hinges on the phenotype; GBM cells with a mesenchymal signature, which inherently have higher baseline p21 levels, show a stronger response than their neurodevelopmental or proneural (PN) counterparts. However, this acts as a double-edged sword for this phenotype: a massive induction of senescence by BMP4 ramps up the risk of relapse fueled by the secretome. The functional need for this protein is absolute: genetically knocking out p21 effectively cancels out the onset of BMP4-induced senescence, clearing away hallmark traits like cellular hypertrophy and SA-β-gal positivity. This confirms p21 as an essential functional regulator of the senescent response in GBM [68]. Against this backdrop, high p21 levels also make cells more sensitive to the senolytic agent Navitoclax, which selectively induces apoptosis in the SA-β-gal-positive fraction, effectively weeding out the p21-overexpressing population and bringing p21 back to normal physiological levels in the surviving cell pool [67,69].

### 4.4. Natural Compounds and Senolytics: Flavonoids and Artesunate

Beyond synthetic inhibitors, the search for senolytic agents has expanded to bioactive natural compounds, with a particular focus on flavonoids and artemisinin derivatives. Among these, Quercetin represents a cornerstone. A naturally occurring polyphenol ubiquitous in fruits and vegetables, Quercetin is one of the first and most validated senolytic candidates [57]. While its specific molecular interference with survival pathways will be detailed in the following section, its strategic importance is highlighted by its frequent use in combination with the tyrosine kinase inhibitor Dasatinib. Preclinical evidence indicates that this combination can favorably remodel the tissue microenvironment. For instance, in animal models, D+Q has been shown to promote the elimination of senescent microglial cells, thereby reducing the recruitment of peripheral monocytes and macrophages and dampening the inflammatory SASP [70]. This suggests that targeting senescent cells with flavonoid-based strategies may exert a therapeutic effect by neutralizing the pro-tumorigenic signals within the microenvironment [71].

In the specific context of Glioblastoma, recent preclinical work has identified other potent senolytic candidates among these molecules:

- **Fisetin:** As demonstrated in the pivotal study by Beltzig et al., this flavonoid exerts significant senolytic activity against TMZ-induced senescence. In LN229 and A172 GBM cell lines, Fisetin treatment significantly reduced the fraction of senescent cells by triggering apoptosis, without inducing toxicity in the proliferating, non-senescent population [72].
- **Artesunate:** Although chemically distinct (a semi-synthetic sesquiterpene lactone derivative of artemisinin), Artesunate showed comparable efficacy in the same study. Already known for its TMZ-sensitizing properties, it induced a significant and selective reduction in the senescent cell burden via apoptosis in GBM models, maintaining a high safety profile [72].

### 4.5. Elucidating the Molecular Mechanisms of Quercetin

**Figure 4:**
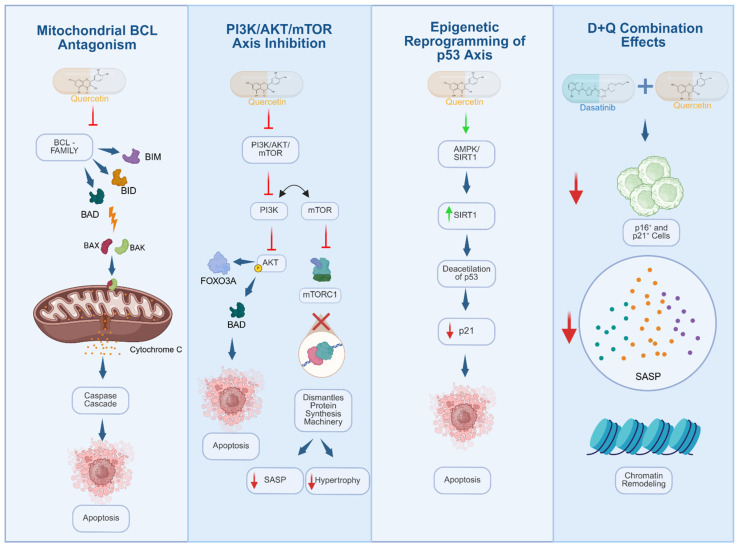
Molecular mechanisms of quercetin and the D+Q senolytic cocktail. Quercetin exerts senolytic effects through several diverse molecular pathways: mitochondrial antagonism of BCL family proteins, triggering the activation of pro-apoptotic proteins (BIM, BID, BAD), which leads to mitochondrial outer membrane permeabilization, cytochrome c release, and initiation of the caspase cascade; inhibition of the PI3K/AKT/mTOR axis, resulting in decreased AKT phosphorylation, modulation of FOXO3A, and downregulation of the mTORC1 complex, thereby reducing protein synthesis, SASP secretion, and cellular hypertrophy; and epigenetic remodeling of the p53 axis via AMPK/SIRT1 activation, which promotes p53 deacetylation and modulates p21 expression. The Dasatinib and Quercetin (D+Q) senolytic combination effectively reduces p16- and p21-positive senescent cells, attenuates the secretion of SASP factors, and induces chromatin remodeling.

The senolytic potency of Quercetin is not attributable to a single target but rather stems from a synergistic assault on three critical survival nodes, displayed in Figure 4: *BCL-2* antagonism, the PI3K/AKT/mTOR signaling axis, and the epigenetic modulation of p53 via SIRT1 (Table 2) [73,74,75].

- **Mitochondrial Disruption and *BCL-2* Antagonism:** At the mitochondrial interface, Quercetin functions as a competitive inhibitor of the anti-apoptotic *BCL-2* family proteins. By neutralizing these guardians, it tips the balance in favor of the pro-apoptotic effectors Bax and Bak, promoting their upregulation and oligomerization [73]. This structural shift compromises the integrity of the outer mitochondrial membrane, triggering the cytosolic release of cytochrome C—an event that serves as the irreversible trigger for the caspase cascade [73,76].
- **Inhibition of the PI3K/AKT/mTOR Axis:** Senescent cells notoriously rely on hyperactive PI3K signaling to sustain their metabolic demands. Quercetin targets this dependency by acting as an ATP-competitive inhibitor of PI3K catalytic subunits and, to a lesser extent, mTOR. This blockade dramatically reduces AKT phosphorylation, unleashing pro-apoptotic targets such as BAD and FOXO3a from inhibitory control while simultaneously suppressing the mTORC1 complex [77,78,79]. Consequently, the protein synthesis machinery required to maintain the hypertrophic, secretory SASP is dismantled.
- **Epigenetic Reprogramming of the p53 Axis:** Finally, Quercetin orchestrates a strategic destabilization of the cell cycle arrest via the AMPK/SIRT1 axis. By upregulating Sirtuin 1 (SIRT1) activity, it induces the deacetylation of p53. This modification dampens p53’s trans activator function on the p21 promoter—a critical maneuver, as p21 is the linchpin holding the senescent state together without triggering death. The resulting drop in p21 levels, combined with the concurrent blockade of *BCL-xl*, forces the cell out of its stable arrest; consequently, unable to repair its accumulated damage or sustain survival signals, the cell collapses into apoptosis [80].

### 4.6. Molecular Mechanisms of Dasatinib and the Dasatinib+Quercetin (D+Q) Combination

Dasatinib is an orally administered tyrosine kinase inhibitor (TKI) [81], initially used for its ability to inhibit kinases such as SRC and EGFR, resulting in the blockade of the pro-proliferative PI3K/AKT/mTOR signaling pathways. Specifically, Dasatinib reduces the phosphorylation of AKT and the ribosomal protein rpS6, downstream markers of PI3K and P70S6K activation, and in combination with temozolomide acts as an autophagy inducer [82]. In various oncological contexts, including hepatocellular carcinoma, Dasatinib exerts an antitumor effect through the inhibition of kinases and their downstream signaling pathways, particularly SFK/FAK and PI3K/PTEN/AKT (Table 2) [83]. In addition to its direct antitumor activity, Dasatinib has gained increasing relevance as a senolytic in the D+Q combination. Clinical and preclinical studies have demonstrated that intermittent D+Q treatment reduces the burden of p16^INK4A-^ and p21^CIP1-positive^ cells, decreases SA-β-gal activity, and significantly attenuates the secretion of SASP factors, including pro-inflammatory mediators and metalloproteinases, as also observed in patients with diabetic kidney disease [84]. More recent evidence also indicates that the D+Q combination does not limit the elimination of senescent cells but can directly impact chromatin organization. In cellular models, repeated D+Q treatment has been associated with a remodeling of nuclear structure and DNA texturing, with a partial normalization of the chromatin characteristics of senescent cells toward a phenotype more similar to that of young cells. These effects are accompanied by modulation of stress-sensitive proteins, such as p53 and p21, and alterations in gene expression, including genes involved in the regulation of SASP [85]. In murine models of ovarian cancer, the D+Q combination has demonstrated senolytic efficacy as an adjuvant to chemotherapy in reducing tumor burden and peritoneal dissemination through the elimination of senescent adipose tissue-derived stromal cells, thus reducing the persistence of a pro-tumor stromal compartment and attenuating the paracrine and metabolic support provided by adipose tissue to ovarian cancer cells [86]. In light of this evidence, the use of D+Q emerges as a promising strategy also in the context of glioma. Within this specific tumor microenvironment, the treatment is highly effective in intercepting critical cellular transitions and niches. Notably, it blocks SRC-driven invasiveness and, especially in combination with Quercetin, dismantles the SASP that fuels the Proneural-to-Mesenchymal Transition (PMT). Furthermore, Dasatinib directly targets Proneural (PN) and hypoxic niches by inhibiting HIF-1α, thereby preventing the hypoxia-induced phenotypic switch and supporting TRAIL-mediated apoptosis [84]. On the clinical front, the Mayo Clinic is currently spearheading a trial looking into a senolytic cocktail—made up of dasatinib, quercetin, and fisetin—paired with temozolomide for patients with previously treated glioma and residual disease. This study sets out to gauge the safety, tolerability, and efficacy of this drug combo, paving the way for its validation in neuro-oncology (NCT07025226).

### 4.7. Elucidating the Molecular Mechanisms of Fisetin’s Senolytic Activity

Recent preclinical breakthroughs have positioned Fisetin (3,3′,4′,7-tetrahydroxyflavone) as a standout senotherapeutic agent. Its efficacy stems from a pleiotropic ability to induce selective apoptosis in senescent cells by simultaneously modulating multiple Senescent Cell Anti-Apoptotic Pathways (SCAPs) [87]. Mechanistically, in these preclinical models, the primary driver of Fisetin’s senolytic action is targeting the PI3K/AKT/mTOR signaling axis, a metabolic super-highway crucial for sustaining the high biosynthetic demands of senescent cells [87,88,89]. Fisetin effectively acts as a metabolic disruptor: it blunts the phosphorylation of AKT and its downstream target, ribosomal protein S6 (a marker of mTORC1 activity); this blockade destabilizes the homeostasis of damaged cells, tipping them toward programmed cell death without compromising the viability of healthy, proliferating tissues which rely less heavily on this hyperactive signaling [87]. Lowering the apoptotic threshold in parallel, Fisetin executes a direct assault on the apoptotic machinery and interferes with *BCL-2* family proteins (such as *Bcl-xL*). By neutralizing these survival guardians, it facilitates the activation of the caspase cascade, ensuring that cells primed for death are successfully eliminated [66]. Through suppression of the SASP and NF-κB blockade beyond direct killing, the flavonoid functions as a potent suppressor of the SASP, which is achieved primarily by blocking the nuclear translocation of the transcription factor NF-κB (p65) and inhibiting the ERK1/2 signaling pathway [90,91]. Crucially, this multifaceted modulation of survival and signaling pathways extends beyond senescent cells to directly impact tumor dynamics. Building upon its robust regulatory profile, Fisetin acts on the phenotypic plasticity of the tumor and it is able to reverse the Glio-Mesenchymal transition (GMT) and the state of acquired TMZ-resistance, acting through the down-regulation of key mesenchymal drivers, including ZEB1 (Table 2) [87].

### 4.8. Therapeutic Value and Senolytic Potential of Artesunate

Artesunate’s therapeutic value stems from its ability to act as a senolytic following TMZ treatment. Specifically, in TIS cells, artesunate dials down RAD51, a key protein in repairing double-strand DNA breaks through homologous recombination (HR). By knocking down RAD51 expression, artesunate cripples the cells’ ability to repair TMZ-induced DNA lesions. This inhibition not only ramps up the alkylating agent’s direct cytotoxicity but also keeps surviving cells from settling into a therapy-induced senescence (TIS) state, ultimately pushing them toward apoptotic cell death [92]. Artesunate also acts on the mesenchymal (MES) component and on the immunosuppressive niche of glioblastoma; by interfering with the STAT3 pathway (the main regulator of the MES phenotype), the compound is able to counteract macrophage polarization towards the M2 phenotype, thus preventing the establishment of a pro-tumoral microenvironment (Table 2) [93]. However, the promising outcomes obtained by the D+Q cocktail and Fisetin across other fields have steered the research focus toward these compounds, effectively ending out Artesunate.

**Table 2:** Senolytic Agents Targeting Anti-Apoptotic Pathways (SCAPs) in GBM.

Compound	Classification	Primary Target(s)/SCAPs	Key Mechanisms of Action	Main Effects in GBM Models	Targeted GBM Subtype	References
**Navitoclax** *(ABT-263)*	Synthetic (*Bcl-2* Family Inhibitor)	*Bcl-2*, *Bcl-xL*, *Bcl-w*	Mimics the BH3 domain to displace pro-apoptotic effectors (Bim, Bid, Bad), triggering MOMP and the caspase 9/3 cascade.	Selectively induces apoptosis in SA-β-gal+ and p21-overexpressing cells; attenuates the pro-tumorigenic niche.	*Mesenchymal (MES-like) and Post-Radiotherapy State:* Exploits the high dependency of radiation-surviving, MES-shifted cells on *BCL-XL* up-regulation.	[67,69]
**Dasatinib**	Synthetic (Tyrosine Kinase Inhibitor)	SRC, EGFR, PI3K/AKT/mTOR, SFK/FAK	Reduces phosphorylation of AKT and rpS6; acts as an autophagy inducer when combined with TMZ.	Reduces p16/p21+ burden and SA-β-gal activity; attenuates SASP secretion; remodels senescent chromatin (often used in D+Q combo).	*Proneural-to-Mesenchymal Transition (PMT):* Highly effective in blocking SRC-driven invasiveness and, especially with Quercetin, dismantling the SASP that fuels PMT.	[57,83]
**Quercetin**	Natural (Bioactive Flavonoid)	*BCL-2* family, PI3K/AKT/mTOR, AMPK/SIRT1	Antagonizes *BCL-2* (promoting Bax/Bak); ATP-competitive inhibitor of PI3K; upregulates SIRT1 to deacetylate p53 (lowering p21).	Triggers caspase cascade; dismantles SASP protein synthesis machinery; forces TIS cells into apoptosis (synergistic with Dasatinib).	*Proneural (PN) & Hypoxic Niches:* Targets hypoxic niches by inhibiting HIF-1α preventing hypoxia-induced phenotypic switch and supporting TRAIL-mediated apoptosis.	[34,73,74,75,76,77,78,79,80,84,94,95]
**Fisetin**	Natural (Bioactive Flavonoid)	PI3K/AKT/mTOR, *BCL-2* (*Bcl-xL*), NF-κB, ERK1/2	Blunts AKT/rpS6 phosphorylation; lowers the apoptotic threshold; blocks nuclear translocation of NF-κB.	Induces selective apoptosis in TMZ-induced TIS cells without harming proliferating cells; acts as a potent SASP suppressor.	*Glio-Mesenchymal Transition (GMT) & TMZ-Resistant State*: Reverses acquired resistance to TMZ and down-regulates key mesenchymal drivers like ZEB1.	[87,88,89,90,91]
**Artesunate**	Semi-synthetic (Artemisinin Derivative)	RAD51 (Homologous Recombination)	Downregulates RAD51, crippling the cell’s ability to repair TMZ-induced DNA double-strand breaks.	Prevents surviving cells from stabilizing into TIS after TMZ treatment, pushing them directly toward apoptotic cell death.	*Mesenchymal (MES) and Immunosuppressive Niche:* Interferes with the STAT3 pathway (a MES master regulator) and counteracts M2-macrophage polarization in the microenvironment.	[87,92,93]

## 5. Clinical Translation of Senolytics: Overcoming Failures and Defining a Therapeutic Model

### 5.1. Understanding the Limitations and Failures of Senolytics in GBM

Although preclinical data are compelling, the clinical translation of senolytics faces significant biological obstacles that explain early therapeutic failures. First, tumor heterogeneity and SCAP redundancy pose a major challenge. Treating a profoundly heterogeneous tumor like GBM with a single-target senolytic, such as the *Bcl-2* inhibitor navitoclax, can be limited by the fact that distinct senescent subpopulations rely on different survival networks; furthermore, GBM cells are known to dynamically reorganize their survival dependencies toward alternative pathways, such as the PI3K/AKT axis or *Mcl-1* upregulation, to evade targeted apoptosis [64]. Second, genetic alterations intrinsic to GBM complicate the response to senescence. Tumors harboring CDKN2A (p16) deletions or TP53 mutations often undergo an incomplete and unstable form of therapy-induced senescence. These cells are highly prone to endoreduplication and senescence escape, rapidly reentering the cell cycle to fuel relapse before senolytics can be effectively administered [45,55].

### 5.2. Advanced Drug Delivery Systems: Overcoming the BBB and Minimizing Off-Target Toxicity

Finally, pharmacokinetic barriers pose a severe restriction. Despite the promising potential of senolytic agents in modulating the glioblastoma tumor microenvironment and counteracting the pro-tumorigenic SASP, their clinical application may be severely limited by poor permeability across the blood–brain barrier (BBB). Dasatinib, for instance, is a well-known substrate of endothelial efflux transporters such as P-glycoprotein and the breast cancer resistance protein; this active efflux drastically limits its accumulation within the brain parenchyma infiltrated by glioma cells, thereby reducing its in vivo efficacy unless the BBB is already compromised [96]. To overcome this translational barrier and ensure that therapeutic concentrations of senolytics reach the tumor site without inducing systemic toxicity, the integration of advanced delivery strategies will be crucial. The use of nanomedicine—such as liposomes or polymeric nanoparticles engineered to exploit receptor-mediated transcytosis (RMT)—represents a promising approach to mask senolytics from BBB efflux mechanisms and enhance their brain tropism [97]. The combination of senolytic therapy with these next-generation drug delivery systems is therefore a necessary step to maximize the eradication of senescent cells in glioblastoma and prevent SASP-driven recurrence. Furthermore, the adoption of targeted drug delivery systems capable of directing therapeutics specifically toward GBM surface markers is essential to minimize off-target toxicity in healthy brain parenchyma. This is particularly relevant because several senolytic targets, such as *BCL-XL* (the primary target of navitoclax), are critical regulators of adult neuron survival; systemic and non-selective inhibition of *BCL-XL* may therefore induce widespread neuronal apoptosis and severe cognitive deficits [98,99].

### 5.3. Therapeutic Timing and Administration Strategies

To overcome initial limitations in clinical translation, current pharmacological perspectives suggest that the timing and route of administration are crucial. Administration of senolytics concurrently with acute radiotherapy or initial courses of TMZ may inadvertently interfere with the physiological DNA damage response (DDR) and the initiation of TIS itself [71]. Therefore, a “one-two hit” strategy appears to be more effective when applied sequentially. Introduction of senolytic adjuvants during the TMZ maintenance phase could strategically exploit the already present senescent cell burden [5,71]. Since senescent cells do not divide, their periodic elimination is sufficient. Consequently, an intermittent, “hit-and-run” administration modality—involving short, high-dose doses over several days, followed by rest periods—is emerging as the preferred approach [59]. This “hit-and-run” strategy is considered crucial: it minimizes systemic toxicity, reduces the risk of adverse effects on healthy tissues, and prevents the emergence of acquired resistance by effectively restoring the inflammatory tumor microenvironment.

### 5.4. Patient Stratification: Toward Biomarker-Guided Senolytic Therapy

The success of senolytics in neurooncology will ultimately depend on rigorous patient stratification, as not all glioblastoma multiforme (GBM) patients will benefit equally from the elimination of senescent cells. Rather than a standardized approach, new preclinical and clinical evidence highlights three critical variables that should guide future senotherapeutic regimens. First, molecular subtyping: The mesenchymal subtype of GBM exhibits intrinsically higher baseline p21 levels, making it significantly more susceptible to p21-dependent senolytic vulnerabilities than proneural subtypes [67]. Second, biomarker monitoring: Patient eligibility should be determined by transcriptomic or immunohistochemical profiling of post-treatment biopsies or liquid biopsies that track circulating SASP factors. Patients showing robust upregulation of p16^INK4A^, p21^CIP1^, and elevated SASP secretomes represent ideal candidates for the “second hit” [11,59]. Finally, age-related variables: Older adult patients possess a significantly higher baseline burden of senescent microglia in the brain parenchyma [100]. Considering age as a stratification factor could help predict the intensity of the pre-existing neuroinflammatory TME, making older demographics particularly relevant for combined approaches using senolytics and neuroprotectives to prevent therapy-induced cognitive decline.

## 6. Conclusions and Future Perspectives

The accumulation of senescent cells in the TME, often resulting from TIS, constitutes a key mechanism for glioma recurrence and therapeutic resistance. Consequently, the senolytic cocktail composed of D+Q has emerged as a promising preclinical strategy for the selective abrogation of senescent glial cells. However, to mitigate the potential neurotoxicity associated with this clearance, melatonin supplementation is proposed as a crucial adjuvant in future translational frameworks. Exploiting its ability to cross the blood–brain barrier [101], Melatonin exerts a dual effect in preclinical models. First, it protects healthy neurons from acute oxidative stress potentially triggered by senolytic clearance by promoting the nuclear translocation of Nrf2, the master regulator of the cellular antioxidant response, and thus stimulating the expression of downstream detoxifying enzymes, such as superoxide dismutase (SOD) and glutathione peroxidase [102]. Secondly, it sensitizes glioma cells to apoptosis by reducing the PI3K/Akt/mTOR axis and suppressing the NF-κB-induced SASP [103]. We hypothesize that this coadministration could act synergistically with D+Q clearance, while preserving the integrity of healthy brain tissue. Alternatively, or potentially in a combinatorial regimen, supplementation with Boswellia Serrata extract, enriched in acetyl-11-keto-β-boswellic acid (AKBA), could offers a distinct neuroprotective paradigm aimed at mitigating the inflammatory sequelae of therapies. While D+Q effectively eliminates senescent glioma cells, the resulting cell lysis inevitably releases free arachidonic acid into the microenvironment. In this scenario, Boswellia acts through its distinctive mechanism as a potent non-redox inhibitor of the 5-lipoxygenase (5-LOX) pathway, an enzyme significantly overexpressed in gliomas to support their proliferation [104]. By blocking this specific cascade, Boswellia prevents the conversion of released arachidonic acid into cytotoxic leukotrienes, effectively neutralizing a potential inflammatory insult to surrounding neurons [105]. This blockade not only synergizes with the pro-apoptotic effects of D+Q, but also critically improves peritumoral cerebral edema and restores the integrity of the blood–brain barrier, providing a neuroprotective shield that could obviate the need for immunosuppressive corticosteroids. Ultimately, adopting this multi-targeted approach, combining senolytic ablation with targeted neuroprotection and immunometabolic modulation, has the potential to overcome the current limitations of resistance to standard treatments. By simultaneously addressing clonal tumor survival and the vulnerability of healthy parenchyma, these integrated strategies could significantly improve the overall prognosis and quality of life in patients with glioblastoma. However, it is imperative to acknowledge that these proposed models currently rely heavily on a strong preclinical rationale. Extensive and rigorously designed clinical trials are now urgently needed to validate whether this biological synergy can effectively and safely translate into clinical practice.

## Figures and Tables

**Table 1:** Differential SASP Profiles and Pro-tumorigenic Effects Across Glial Populations Following Therapy-Induced Senescence (TIS).

Cell Type	Key SASP Factors	Primary Pathways & Mediators	Impact on TME & Tumor Progression	References
**Senescent Astrocytes (SnAs)**	CXCL12, G-CSF, TNF-α, sICAM-1, IL-6, HGF	c-Myc–Max complex (activated by TNF-α) HGF/Met RTK interaction	Fosters an immunosuppressive TME (recruitment of CXCR2+ cells); promotes tumor growth, migration, and invasion.	[5]
**Oligodendrocyte Precursor Cells** **(OPCs)**	EGF, FGF1 (Upregulated) TGF-β (Downregulated)	Modulation of physiological myelination signaling TGF-β signaling downregulation	Modulates chemoresistance; reshapes tumor vasculature and the microglial landscape.	[41]
**Microglia**	IL-6, IL-8, CCL2, TNF-α, TGF-β	NF-κB activation MAPK pathways	**Acute:** Induces neurotoxicity and neuronal apoptosis. **Chronic:** Creates an immunosuppressive niche, protecting the tumor from immune surveillance.	[41]

## Data Availability

No new data were created or analyzed in this study.
